# Design and Experimental Validation of a Multiple-Frequency Microwave Tomography System Employing the DBIM-TwIST Algorithm

**DOI:** 10.3390/s18103491

**Published:** 2018-10-16

**Authors:** Syed Ahsan, Ziwen Guo, Zhenzhuang Miao, Ioannis Sotiriou, Maria Koutsoupidou, Efthymios Kallos, George Palikaras, Panagiotis Kosmas

**Affiliations:** 1 Faculty of Natural and Mathematical Sciences, King’s College London, Strand, London WC2B 4PH, UK; zhenzhuang.miao@kcl.ac.uk (Z.M.); ioannis.sotiriou@kcl.ac.uk (I.S.); maria.koutsoupidou@kcl.ac.uk (M.K.); 2 MediWise Ltd., London E1 2AX, UK; themos.kallos@mediwise.co.uk (E.K.); george.palikaras@mediwise.co.uk (G.P.)

**Keywords:** printed monopole antenna, wideband, array, microwave tomography, DBIM, medical imaging

## Abstract

We present a first prototype of a wideband microwave tomography system with potential application to medical imaging. The system relies on a compact and robust printed monopole antenna which can operate in the 1.0–3.0 GHz range when fully immersed in commonly used coupling liquids, such as glycerine–water solutions. By simulating the proposed imaging setup in CST Microwave Studio, we study the signal transmission levels and array sensitivity for different target and coupling liquid media. We then present the experimental prototype design and data acquisition process, and show good agreement between experimentally measured data and results from the CST simulations. We assess imaging performance by applying our previously proposed two-dimensional (2-D) DBIM TwIST-algorithm to both simulated and experimental datasets, and demonstrate that the system can reconstruct simple cylindrical targets at multiple frequencies.

## 1. Introduction

Microwave tomographic (MWT) methods for medical imaging estimate the spatial distribution of dielectric properties in a tissue region by solving an electromagnetic (EM) inverse scattering problem [1,2]. The dielectric properties of tissues have been extensively studied and reported in the literature; see, for example, the early studies in [3,4]. Reconstructing the dielectric profile of a region inside the human body to detect a pathological condition exploits the possibility of a significant dielectric contrast between healthy and disease-affected tissues. In the case of breast cancer, for example, the cancerous tissue contrast depends on the density of the healthy tissue surrounding the tumor. The malignant tumor contrast can range from 10:1 for adipose to 10% for dense fibroglandular tissue [5]; in the latter case, the use of suitable, nontoxic contrast agents [6] could provide a means of detection based on a differential imaging approach [7].

MWT techniques for medical imaging have been applied primarily to breast imaging, and their use for detection and imaging of stroke are also attracting the interest of many groups worldwide [8,9]. Experimental prototypes have been developed and some clinical trials have been carried out [10,11,12,13,14,15]. Imaging prototypes using radar-based methods have also been reported, e.g., [16,17,18,19,20]. The first MWT prototype reported in clinical trials [10] consisted of an array of monopole antennas immersed in a lossy liquid, such as a glycerine–water solution. These long monopoles are simple to construct and model in the MWT algorithm, but operate in a limited bandwidth and produce surface wave propagation and multipath signals, thus requiring a high loss immersion medium to improve data quality [21]. The MWT system in [22] also uses narrowband, waveguide antennas operating at 900 MHz, which are immersed in a water solution of varying degrees of salt concentrations. Wideband systems have also been proposed but mostly at higher frequencies, such as the system of 24 co-resident Vivaldi antennas operating between 3.0 and 6.0 GHz presented in [12]. More recently, a portable wideband system in the range 0.75–2.55 GHz was proposed for intracranial hemorrhage detection [23].

Our aim to design a wideband MWT system in the 1.0–3.0 GHz range is driven by our previous numerical studies for microwave breast imaging with anatomically complex numerical breast phantoms [24,25,26]. These studies have demonstrated that reconstruction stability and accuracy are greatly enhanced by using a low-frequency reconstruction (e.g., at 1.0 GHz) as a prior step to reconstructing data at higher frequencies. To achieve this goal experimentally, we are proposing in this paper a new MWT system comprising compact printed monopole antennas which can operate in 1.0–3.0 GHz. In addition to this excellent bandwidth for MWT applications, these printed monopole antennas have a very small size (12×15 mm^2^) which can reduce unwanted multipath signals relative to conventional monopoles [21], while their monopole-resembling operation can be modelled more accurately by our imaging algorithm relative to more complex antenna designs [12].

The remainder of the paper is organized as follows: Section 2 presents methodologies and results for the design, data acquisition, and sensitivity of the proposed MWT system. Section 3 focuses on system validation, first by comparing signals from simulation and experiment, and then by presenting inversion results to confirm the ability of the system to image canonical targets, using our previously developed DBIM-TwIST algorithm. Finally, Section 4 presents some concluding remarks and future steps for the system.

## 2. Materials and Methods

### 2.1. Antenna Design and Characterization

Designing a small and efficient antenna is very important in microwave tomography, as a smaller size can accommodate a greater number of antennas in the array. Employing multiple antennas in the system is helpful in acquiring sufficient data to deal with the ill-posedness of the inverse problem, but also poses a cross-coupling issue between the constituting antenna elements of the array. A lossy immersion medium can minimize cross-coupling and multipath signals [21,27,28], and at the same time broaden the antenna’s operation bandwidth and improve impedance matching with human tissue, such as skin. There is, however, a trade-off between these benefits and the attenuation that a lossy immersion liquid will cause to the signals received by the imaging system. For these reasons, the choice and impact of immersion and coupling media on the design and performance of microwave tomography systems have been extensively studied in the literature [29,30,31,32].

**Figure 1:**
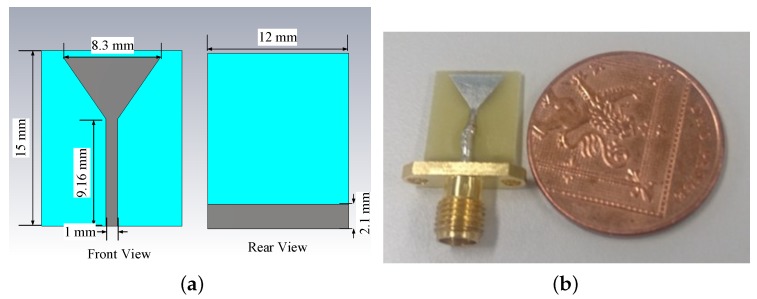
(**a**) Schematic of the proposed antenna modelled in CST Microwave Studio; (**b**) Photo of the fabricated antenna.

Glycerine–water mixtures are attractive candidates, as they are easy to make and allow control of the mixture’s dielectric constant and conductivity [27]. Corn syrup mixtures with water can produce the same range of permittivity values as glycerine–water mixtures, but with quite lower conductivity [32]. The dependence on frequency, however, is stronger, and the solution itself is too viscous and poses handling issues. Based on these considerations, we designed a compact antenna that can operate in the 1.0–3.0 GHz frequency range when immersed in coupling media commonly used in microwave tomography. Figure 1a shows a schematic of the front and rear views of the printed monopole antenna modelled in CST Microwave Studio. The dimensions of the antenna patch, substrate, transmission line, and the partial ground are included in the drawing. The printed monopole antenna has been realized on FR-4 substrate and is fed with an SMA connector. A fabricated prototype is shown in Figure 1b.

**Figure 2:**
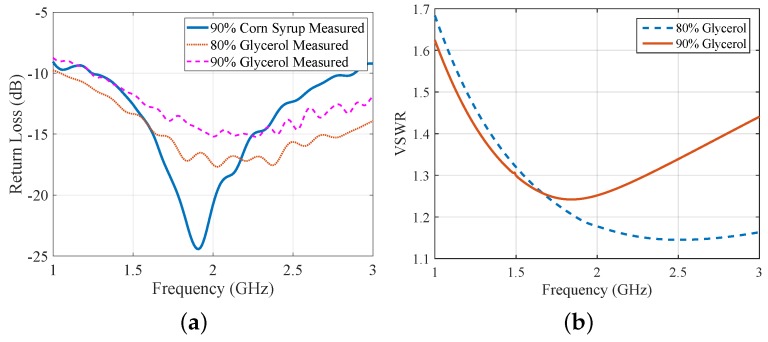
(**a**) $S_{11}$ for the printed monopole of Figure 1 when immersed in the glycerine and corn syrup mixtures measured in Figure 3; (**b**) Calculated voltage standing wave ratio (VSWR) of the antenna immersed in 80% and 90% glycerine–water mixtures, using CST simulations.

**Figure 3:**
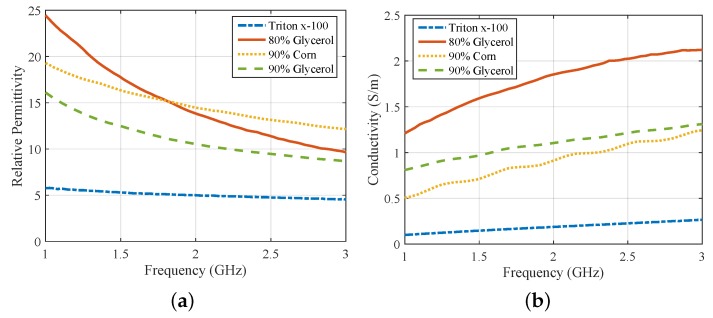
Measured (**a**) relative permittivity and (**b**) conductivity for the immersion liquids considered in this study (material properties of Triton X-100 were measured at Politecnico di Torino).

The antenna’s measured reflection coefficient $S_{11}$ is plotted in Figure 2a for three different immersion liquids. A value under −10 dB for $S_{11}$ suggests good operation in the whole range of interest, 1.0–3.0 GHz. Moreover, Figure 2b plots the voltage standing wave ratio (VSWR) inside 80% and 90% glycerine–water immersions, as calculated by CST simulations. The VSWR of the antenna stays below 2.0 in 1.0–3.0 GHz, thus demonstrating good impedance matching for the entire frequency range of interest. The dielectric properties of the three immersion liquids considered in Figure 2a were measured using Keysight’s dielectric spectroscopy kit, and are plotted in Figure 3. In addition, we measured the properties of Triton X-100, which is a low-loss reference liquid that has been proposed and studied for microwave imaging [33]. We then imported the measured data into CST Microwave Studio, using second-order curve fitting to model frequency dispersion. Figure 3 plots the dielectric properties for the four materials after curve fitting the experimentally measured dataset.

### 2.2. Array Setup and Data Acquisition Process

**Figure 4:**
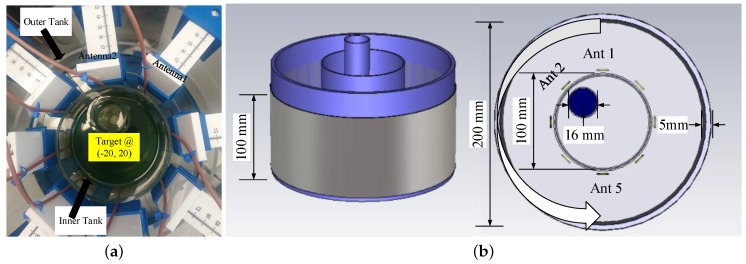
(**a**) Photo of the hardware system prototype used in our measurements; (**b**) Schematic of the imaging system hardware modelled in CST.

The actual experimental system is presented in Figure 4a, while a drawing of the setup is given in Figure 4b. The setup consists of two concentric cylindrical tanks with 100 and 200 mm diameters, respectively. A target of 16 mm diameter can be placed inside the inner tank to emulate the discontinuity in the homogeneous background medium. We surrounded the outer periphery of the larger tank with an absorber (ECCOSORB MCS) and covered it with a metallic shield to reduce surface waves propagating along the periphery of the immersion tank, as well as any external interference. Our eight-antenna configuration forms a circular ring inside the outer acrylic tank of Figure 4a, as also shown in more detail in the schematic of Figure 4b. We note that the choice of eight antennas was made to simplify data acquisition; from a theoretical point of view, this number should be equal to 2βα, where β is the wave number and α is the radius of the imaging domain [34]. Vertical and horizontal mounts allow us to control the antenna positions with good precision and vary their height as well as the array diameter, which was kept at 130 mm in the present experiments.

To overcome the absence of a switch matrix in the present setup, we collected bistatic data from a two-port vector network analyzer (VNA) from Keysight, and acquired synthetic data for the array by switching manually the receiver to each of the seven positions in Figure 4. The measurement process included the following steps: First, we assembled the setup and calibrated the VNA set to 201 data points with an averaging factor of 5 and IF bandwidth 200 Hz. Then, we filled up the immersion liquid in the tanks, and we connected the transmitter and receiver to the VNA’s Port1 and Port2, respectively. We then changed the receiving antenna at Port2, and after a complete sweep for the seven positions, we moved the transmitting antenna to the next position and repeated the process. We then introduced the target inside the imaging domain and repeated the process for the ‘with target’ case, which produces the scattered signal information to be used by the algorithm for reconstruction.

### 2.3. System Sensitivity Evaluation Using Numerical Simulations

We evaluated the performance of our imaging system shown in Figure 4 by modelling in CST Microwave Studio. We considered two sets of simulations for this purpose; the first set studies signal propagation inside the target-free tank filled with one of the immersion liquids, while the second also includes a cylindrical target with various contrasts. The target-free simulations allow us to compare the level of transmitted signals for the different immersion liquids in Figure 3, which should be above the noise of the VNA used in our measurements. To study the system’s response to a target, we calculated the signal scattered from a 16 mm diameter cylindrical target immersed in the 90% glycerine–water mixture. To assess the effect of varying the target contrast to the received signals, we also compared the signal from the water target to those of a metallic target modelled in CST as a perfect electric conductor (PEC), as well as a salt–water mixture.

**Figure 5:**
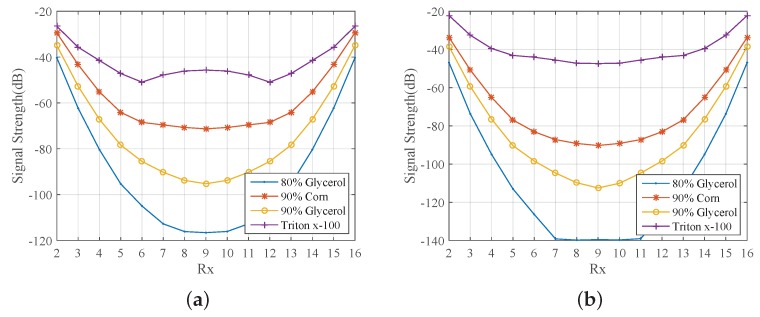
CST-calculated received signal strength as a function of receiver number for a 150 mm diameter array immersed in 80% glycerine, 90% corn syrup, 90% glycerine, and Triton X-100, when Transmitter 1 transmits (**a**) at 1.5 GHz and (**b**) 2 GHz.

To better sample the signal levels transmitted in the tank filled with one of the immersion liquids considered in this study, we first simulated a 16-antenna array with a larger, 150 mm diameter. Figure 5 plots the received signal strength at 1.5 and 2.0 GHz as a function of receiver number, where receivers are numbered in the same order as in Figure 4b. Comparison of the two plots shows the strong level of attenuation that the signals experience as the frequency increases. As expected, we observe that the maximum signal strength is received when (low-loss) Triton X-100 is used as the background medium. The signal level for 80% glycerine–water is very low, below −140 dB at 2.0 GHz, for example, but is considerably higher for the 90% mixture. The 90% corn syrup shows higher transmission levels than both glycerine mixtures, but is more difficult to handle experimentally (as is the Triton X-100).

**Figure 6:**
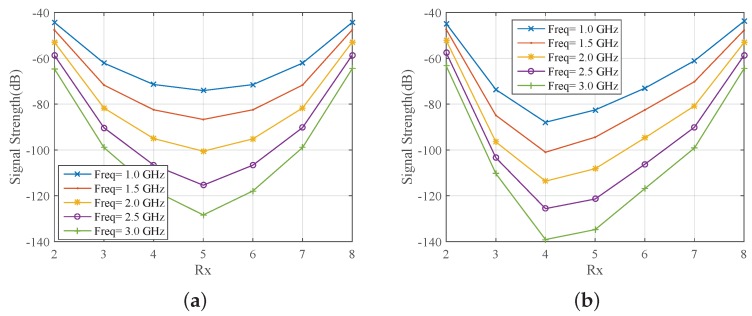
CST-calculated received signal strength as as function of receiver number when the array is immersed in 90% glycerine–water (**a**) without target and (**b**) with metallic (perfect electric conductor, PEC) target.

The results of Figure 5 suggest that we can use a 90% glycerine–water mixture as our system’s immersion liquid if we wish to avoid handling issues with corn syrup and Triton X-100. We thus proceeded with simulating our exact measurement setup comprising an eight-antenna array of 130 mm diameter filled with the 90% glycerine–water mixture. The received signal strength is plotted as a function of receivers for every 500 MHz in Figure 6a. The same information is plotted in Figure 6b, when we introduce a strong PEC scatterer to validate the imaging system’s sensitivity for the given target size. As expected, the curves of the received signal strength are not symmetric any longer, and the impact on the received signals correlates with the target’s location shown in Figure 4b. In particular, the signal strength is visibly reduced at receivers for which the transmitted signal is “blocked” by the PEC scatterer.

**Figure 7:**
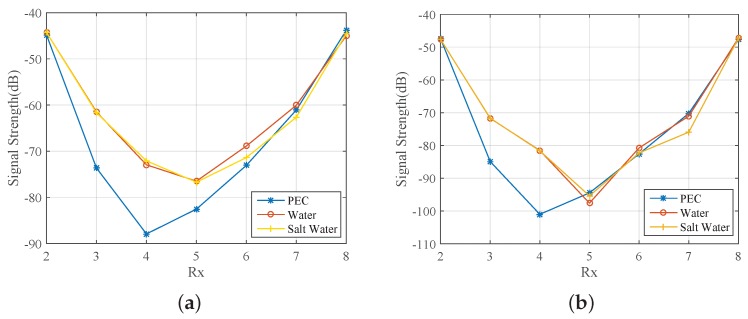
Comparison of the impact of a metallic (PEC), water, and salt–water cylindrical target when the array is immersed in 90% glycerine–water: Received signal strength as a function of receiver number (**a**) at 1.0 GHz and (**b**) at 1.5 GHz.

**Figure 8:**
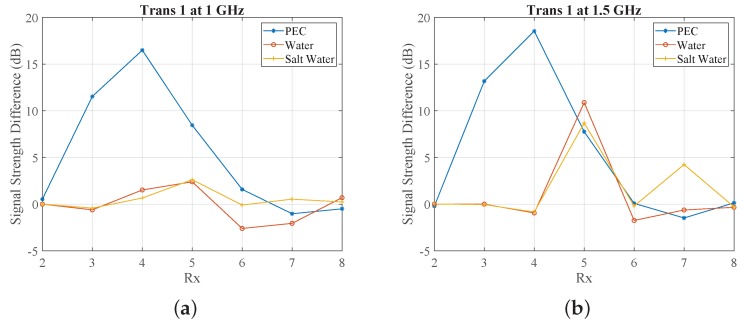
Differences in dB of received signal strength (No target–with target) as a function of receiver number when the array is immersed in 90% glycerine–water with metallic (PEC), water, and salt–water targets (**a**) at 1 GHz (**b**) at 1.5 GHz. Note that positive values in dB result from received signals that are reduced in the presence of the target.

In line with our interest in microwave medical experiments, we introduced in our setup two different liquids for our target: water and a salt–water mixture (10 mg/mL concentration). These targets exhibit high contrast relative to the 90% glycerine–water background. Figure 7 compares the impact of the PEC with these two dielectric targets on the received signal strength at two representative frequencies, 1 GHz and 1.5 GHz. We observe very similar signal levels and trends for the water and salt–water targets, which are of course different from the PEC target which blocks the signal in the opposite antennas. We also calculated and plotted (in dB) ratios of the received signal strength with and without the target in Figure 8. While this difference is considerably stronger for the PEC target, it is also quite evident for the dielectric targets, for which the most notable difference is in the receivers across from the targets. These signal differences are not straightforward to interpret, as they are caused by various interactions of the transmitted signal with the system and target. However, a tomographic approach which can model these interactions as faithfully as possible should be able to reconstruct these targets using either numerical or experimental data, provided that measurement errors do not dominate the experiments.

## 3. Results

### 3.1. Comparison of CST and Experimental Results

**Figure 9:**
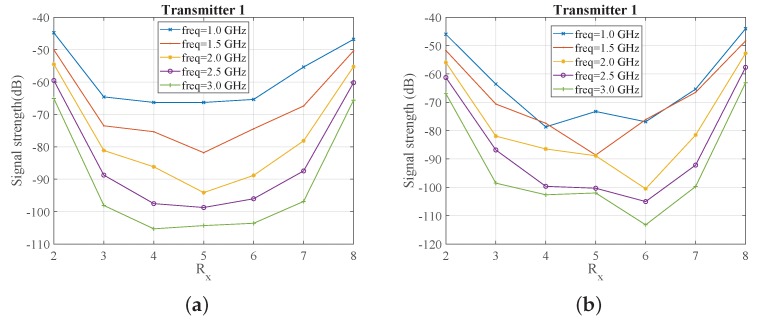
Received signal strength as a function of receiver number when the array is immersed in 90% glycerine–water for the experimental setup: (**a**) no target and (**b**) water target.

Our experimental measurements first acquired “target-free” data of the tank filled with 90% glycerine–water. We then introduced a 16 mm target tube filled firstly with water and then with a salt–water mixture (10 mg/mL). Figure 9 depicts the received signal strength with and without the target as a function of the receivers for the first antenna transmitting. Comparing Figure 6a and Figure 9a, we observe a very good agreement between CST simulations and experimental measurements for the target-free case. Differences can be attributed to factors that were not taken into account in the CST simulations, such as coupling between the cables, instrument noise, and interference from environmental factors. We note that the noise floor for our used VNA settings is −100 dB, and, therefore, signals below this value are susceptible to noise.

**Figure 10:**
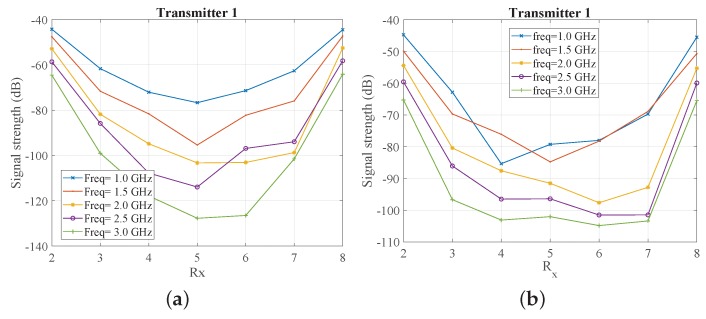
Received signal strength as function of receiver number when the array is immersed in 90% glycerine–water and salt–water target is introduced: (**a**) CST simulation; and (**b**) experimental prototype.

**Figure 11:**
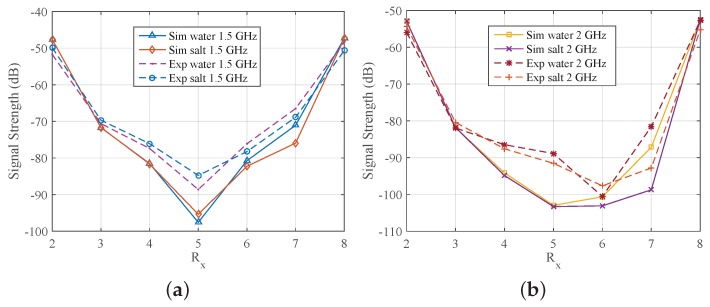
Comparison of received signal strength as a function of receiver number from simulations and measurements when the array is immersed in 90% glycerine–water, and targets are introduced. Plots at (**a**) at 1.5 GHz and (**b**) at 2 GHz.

We also assessed the impact of the target on experimentally measured signal propagation, in a similar manner to the CST simulation studies. Results are shown in Figure 9b and Figure 10b, which plot the same data as in Figure 9a for the water target and salt–water mixture (10 mg/mL), respectively. Results from CST simulations for the salt–water target are shown in Figure 10a. Trends observed in both cases are very similar, except at 1.0 GHz where the experimental data is significantly different from the simulation results. The antenna operates less efficiently at 1.0 GHz, and we observed that the coupling between the cables causes a significant discrepancy between experimental and simulation data at lower frequencies. For frequencies where the antenna is more efficient, such as 1.5 and 2.0 GHz, there is good agreement between CST simulations and measured data, as shown in Figure 11.

### 3.2. Reconstructions Using the DBIM-TwIST Algorithm

The observed differences in received signal strength plots due to the target suggest that there is potentially sufficient contrast for an inverse scattering algorithm to reconstruct the target. To test this hypothesis and validate our proposed wideband microwave tomography system, we applied our previously developed DBIM-TwIST algorithm to the data from our experimental measurements as well as CST simulations. The DBIM-TwIST algorithm has been analyzed and validated with numerical microwave breast imaging simulations in previous work [24,25]. It is based on the distorted Born iterative method (DBIM), which solves the nonlinear scattering problem iteratively by applying a Born approximation at each iteration. The solution to the resulting linear problem is used to update the background medium recursively, until the data residual converges to a minimum [35]. In the DBIM-TwIST algorithm, the linear problem at each DBIM iteration is solved by our own optimized implementation of the two-step iterative shrinkage-thresholding algorithm [24]. The algorithm’s forward solver is based on the finite-difference time-domain (FDTD) method, which is applied to a simplified two-dimensional (2-D) model of the tank with line sources at the same locations as the monopoles. The FDTD algorithm is excited with a Gaussian pulse operating in the 0.5–3.5 GHz range, and uses a single-pole Debye model [35] to capture the dispersion of the 90% glycerine–water mixture. To calibrate data from our forward model with CST or experimental data, we apply a standard calibration procedure using the “empty tank” datasets [31].

**Figure 12:**
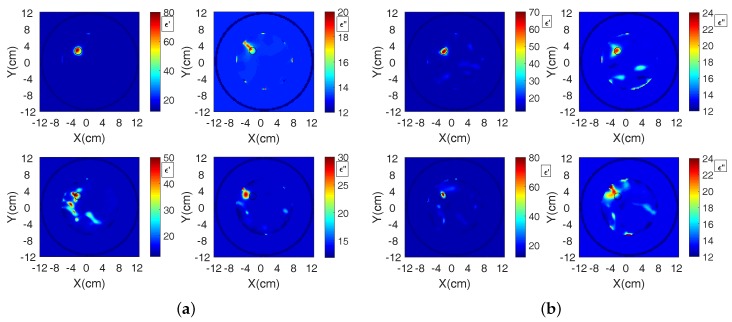
Reconstructed real and imaginary parts of the complex permittivity for the region inside the antenna array of Figure 4 in the presence of: (**a**) water and (**b**) salt–water targets. The top row corresponds to data calculated from CST simulations and the bottom row to data measured from our experimental system. The complex permittivity was calculated at 1.2 GHz from the Debye parameters, which were reconstructed using a frequency hopping approach at 1.2, 1.5, 1.8, 2.1, and 2.4 GHz.

Figure 12 presents complex permittivity reconstructions using the data obtained from simulations and measurements. The images depict the whole tank; however, the reconstruction domain is a ring with a diameter of 120 mm, which is concentric and at 10 mm distance from the antenna array to avoid imaging artifacts from the antennas. Data were collected at the eight transceivers in our system of Figure 4 for both target media, i.e., water and the salt–water mixture. For all reconstructions, cubic voxels of 2.0 mm sides were used in our imaging algorithm. We applied the DBIM-TwIST algorithm with a frequency hopping approach at 1.2, 1.5, 1.8, 2.1, and 2.4 GHz, and performed 20 iterations at each frequency. At the start of the inversion process at 1.2 GHz, the imaging domain was filled with the background glycerine–water medium of known properties. The reconstructed properties at 1.2 GHz were then inserted as the initial guess for the reconstruction at 1.5 GHz, and so on. The total computation time for the forward solver for all eight antennas was 7 s per iteration, and the inversion time was 0.5 s, leading to a total run time of 7.5 s for each DBIM iteration. The Matlab code was run on an Intel i7 3.4 GHz processor with 16 GB RAM memory.

The actual real and imaginary permittivity values of the water and salt–water targets at 1.2 GHz were measured as $\epsilon^{\prime }=78.6,\epsilon^{\prime \prime }=4.8$, and $\epsilon^{\prime }=77.5,\epsilon^{\prime \prime }=30.5$, respectively. The respective values for the background glycerine–water mixture were $\epsilon^{\prime }=14.2,\epsilon^{\prime \prime }=13.3$. Both targets, therefore, have a strong contrast with respect to the background, which makes quantitative imaging challenging within the Born approximation. The results of Figure 12, however, are encouraging. For the CST data used in the top row, the target is detected and localized, and the estimated values are close to the actual, except for the imaginary part of the water target. Interestingly, differences in the estimated values for the water and salt–water targets correlate with differences in the actual values (the real part is higher, and the imaginary part is lower for water relative to salt–water). There are of course also errors and artifacts in these images, which are more pronounced in the bottom row which reconstructs the experimental data. Measurement errors in these cases do not allow the same accuracy as CST data, but reconstructions are not far from the actual values, especially in the case of the salt–water target.

**Figure 13:**
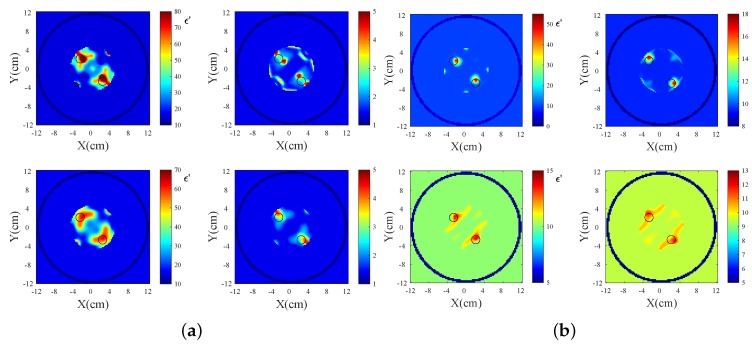
Reconstructed real and imaginary parts of the complex permittivity for the region inside the antenna array of Figure 4 in the presence of two water-filled targets. Data are generated from CST simulations. The top row corresponds to the use of the TwIST solver, and the bottom to the use of the CGLS solver. The figure shows single-frequency reconstructions at (**a**) 1.0 GHz and (**b**) 2.2 GHz.

We note that the ability of our algorithm to reconstruct accurately more complex targets, such as the breast interior, has been demonstrated in [25], but using data from FDTD simulations. The reconstructions presented in this paper, on the other hand, aim to investigate reconstruction quality using data from the particular MWT system. To examine the system’s ability to image a more complex scenario, we added a second, identical target in the CST model, placed symmetrically in the third quadrant of the imaging domain (near Antenna 5 in Figure 4). Single-frequency reconstructions of the two water-filled targets at two representative frequencies are presented in Figure 13. The top row images resulted from using the DBIM-TwIST algorithm, while the TwIST was replaced with CGLS in the bottom row images.

These images suggest good performance for this more complex imaging scenario of two targets. They also demonstrate the impact of the inverse solver in the reconstructions; at 2.2 GHz, the TwIST solver can resolve the two targets more accurately, but, at 1.0 GHz, it overestimates the complex permittivity values and results in strong artefacts in the imaginary part. The reconstruction domain was a ring with a diameter of 100 mm for these reconstructions, but we are again showing images of the whole tank. We also note that these reconstructions have not been optimized by tweaking the TwIST solver’s parameters or using frequency hopping [24,25]. This will be the topic of future work, where more complicated and realistic imaging scenarios will be introduced for the proposed MWT system.

## 4. Discussion

We present a design and preliminary investigation of a wideband MWT system, which was based on small, custom-made printed monopole antennas. These antennas operate efficiently between 1.0 and 3.0 GHz when immersed in liquids commonly used in microwave imaging. We also assessed the signal strength received by our array using CAD-based 3-D CST simulations of our system, and introduced two different dielectric targets (water and a water–salt mixture) to study numerically their effect on the recorded signal strength. We chose an eight-antenna array for our current experimental setup, and present results that show good agreement between simulations and experimental measurements. Finally, we used our in-house DBIM-TwIST algorithm to reconstruct the images from the acquired data, and show that we can successfully reconstruct the considered dielectric targets.

The proposed system and reconstruction method differentiate this work significantly from previously proposed MWT systems, such as those in [10,11,12,13]. First, the hardware allows the use of a wide range of frequencies, 1.0–3.0 GHz, which is critical in order to guarantee robustness and enhanced resolution for the DBIM-TwIST algorithm [25]. Moreover, using printed monopoles allows a simple modelling of the antennas as point sources by the forward solver of our algorithm. More importantly, the DBIM-TwIST approach can produce images of enhanced resolution relative to approaches based on traditional solvers, such as the CGLS (see Figure 13). On the other hand, limitations of the present work include having considered a cylindrical geometry without variations along the height of the tank, and a data acquisition process without a multi-port system, which is prone to long data acquisition times and experimental errors.

To overcome these limitations, our ongoing and future work will refine the data acquisition and inversion process further, and assess the system’s imaging abilities for various different experimental scenarios. Our next prototype will feature a multi-port system and multiple antenna rings with a minimum of 12 antennas for each cylindrical ring, which is closer to the optimal number, as argued in [34]. We will also perform experimental and numerical studies to assess the system’s ability to reconstruct “weak” targets with a small contrast relative to the background medium, as well as investigate more complicated imaging scenarios which are relevant to realistic medical imaging problems. Ultimately, our goal is to create a prototype that could be used in medical imaging applications of interest [36], such as cancer or stroke detection and treatment monitoring.
